# Se Nanoparticles Induce Changes in the Growth, Antioxidant Responses, and Fruit Quality of Tomato Developed under NaCl Stress

**DOI:** 10.3390/molecules24173030

**Published:** 2019-08-21

**Authors:** Mónica Carolina Morales-Espinoza, Gregorio Cadenas-Pliego, Marissa Pérez-Alvarez, Alma Delia Hernández-Fuentes, Marcelino Cabrera de la Fuente, Adalberto Benavides-Mendoza, Jesús Valdés-Reyna, Antonio Juárez-Maldonado

**Affiliations:** 1 Maestría en Ciencias en Horticultura, Universidad Autónoma Agraria Antonio Narro, Saltillo COA 25315, Mexico; 2 Departamento de Síntesis de Polímeros, Centro de Investigación en Química Aplicada, Saltillo COA 25294, Mexico; 3 CONACyT-Instituto Mexicano del Petróleo, Ciudad de México 07730, Mexico; 4 Instituto de Ciencias Agropecuarias, Universidad Autónoma del Estado de Hidalgo, Tulancingo HID 43600, Mexico; 5 Departamento de Horticultura, Universidad Autónoma Agraria Antonio Narro, Saltillo COA 25315, Mexico; 6 Departamento de Botánica, Universidad Autónoma Agraria Antonio Narro, Saltillo COA 25315, Mexico

**Keywords:** nanotechnology, abiotic stress, antioxidants, lycopene, β-carotene, human health

## Abstract

Nanotechnology represents an opportunity to improve the use of elements in agriculture. Selenium is an element that is beneficial to plants and essential to the human diet. The size of nanoparticles gives them characteristics that can enhance the benefits that selenium provides to plants. The objective of the present study was to determine the effects of selenium nanoparticles on the growth, antioxidant responses, and fruit quality of tomato developed under NaCl stress. Four doses of selenium nanoparticles (1, 5, 10, and 20 mg L^−1^) under NaCl stress, only NaCl, and a control were evaluated. The results showed that the impact of salinity on the growth of the tomato crop can be reduced with the application of selenium nanoparticles. However, the amount of both enzymatic and non-enzymatic compounds significantly increased in the leaves and fruits of tomato. The results suggest that the application of selenium nanoparticles generated a positive effect against salinity in the tomato crop; moreover, it had a positive impact on the content of beneficial biocompounds for human health in tomato fruits.

## 1. Introduction

Salinity is one of the environmental factors that affect crop productivity in addition to being one of the most widely distributed conditions in the world [1]. This factor directly affects the assimilation of other nutrients, and can also directly generate toxicity in crops, which makes it even more dangerous for crop production [2,3]. Under the effects of salinity, plants may undergo modifications that can be morphological, such as a reduction in the foliar area, or biochemical, such as adjustments to its osmotic potential [4]. In addition, overproduction of reactive oxygen species occurs [5], which can cause damage to membranes, proteins, lipids, and even DNA [6]. Because of this, plants produce different enzymatic and non-enzymatic antioxidants to defend against oxidative stress.

Fortunately, nanotechnology has generated promising results in different areas of science, including agriculture [7]. Therefore, its application in agriculture can be a viable option, especially the use of nanoparticles (1–100 nm particles), since they are similar to biological molecules such as proteins, and are able to pass through cell membranes [8]. Due to their small size, they have physical and chemical properties that differ significantly from the usual ones that particles on a larger scale have [9]. The application of several types of nanoparticles (NPs) has been evaluated in different crops with results that vary according to the species, dose, and type of NPs [10]. In some cases, low concentrations of NPs (less than 100 ppm applied to soil, foliage, or seeds) have generated effective responses of induction and accumulation of certain metabolites or phytochemicals [5,11], which can be antioxidants or defense compounds.

Selenium is essential to human health due to the fact of its role as a cofactor of enzymes related to antioxidant metabolism [12]; however, in plants, Se also exerts a positive effect on antioxidant capacity [13]. It has been shown that selenium nanoparticles (Se NPs) can function as stimulants in plants, improving their antioxidant defense system and, therefore, their ability to tolerate stress [14]. Selenium NPs also have excellent biological properties and low toxicity [15], unlike the bulk form that has a well-documented toxic effect (due to the oxidative stress) in plants at high concentrations [16]. For example, the application of selenate at 10 mg L^−1^ was found to have negative effects on the photosynthetic capacity and growth of *Nicotiana tabacum*, while in the form of nanoparticles in higher concentrations (100 mg L^−1^), no negative effects were observed [17]. Therefore, its application to crops may be a good option, and, due to the fact of its properties as a consequence of its size, better results than those observed in bulk can be expected. However, there is a lack of information on the use of Se NPs and their effect on tolerance to abiotic stress, and there is no information about Se-NP-induced changes in the antioxidant compounds. This information is very relevant, since the production of bioactive compounds in plants for human consumption, such as tomatoes, could positively influence human health.

Considering the potential positive effects of Se NPs, the objective of the present investigation was to analyze changes in the crop growth, antioxidant responses, accumulation of bioactive compounds, and fruit quality of tomato when plants are grown under conditions of salt stress and with an application of selenium nanoparticles.

## 2. Results

### 2.1. Crop Growth

**Figure 1:**
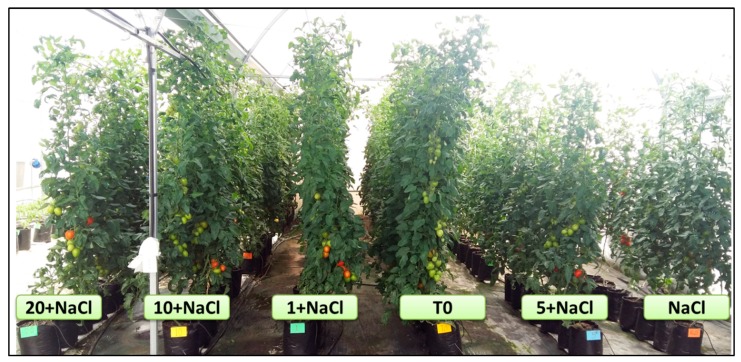
A tomato crop under NaCl stress and an application of Se NPs. T0: Control. NaCl: NaCl-only treatment. 1, 5, 10, and 20 + NaCl: Represent the amount of Se NPs applied (mg L^−1^) plus 50 mM of NaCl.

With the exception of the number of clusters, in the rest of the evaluated variables significant differences were observed among the treatments (Table 1). Except in the number of leaves, in all variables, the control showed better results than the rest of the treatments. The number of leaves was greater in the NaCl-only treatment, surpassing the control and the treatment with 10 mg L^−1^ of Se NPs + NaCl by 4.8% and 4.1%, respectively. As for the weight of fruits per plant, treatment with 10 mg L^−1^ of Se NPs + NaCl was better than the NaCl-only treatment by 12.8%. Also, in fresh weight of aerial biomass, the same treatment was better than the NaCl-only treatment by 12.3%. Considering the effect of the treatments on all of the variables, the results indicate that the control was the best. However, the second-best treatment was 10 mg L^−1^ of Se NPs + NaCl, which had a positive influence on fruit weight and aerial biomass. It is important to note that no damage was observed due to the toxicity of the Se NPs; the crop developed “normally” as can be seen in Figure 1.

### 2.2. Photosynthetic Pigments

**Figure 2:**
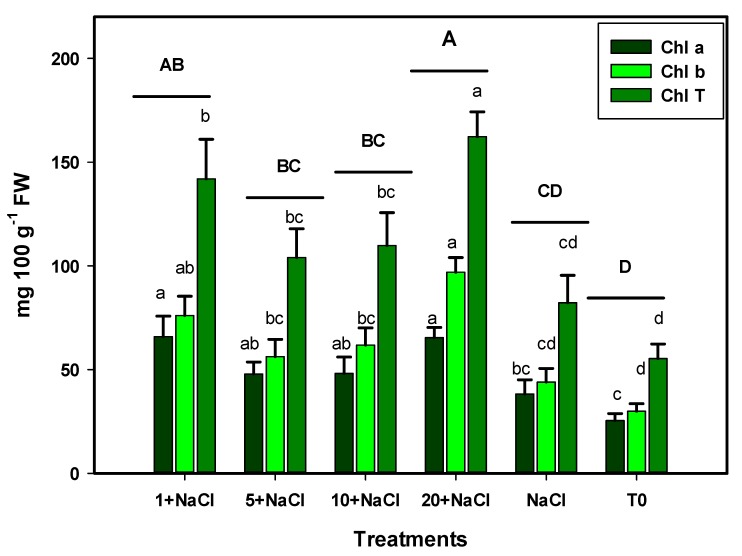
Photosynthetic pigments in leaves of tomato plants under NaCl stress and an application of Se NPs. T0: Control. NaCl: NaCl-only treatment. 1, 5, 10, and 20 + NaCl: Represent the amount of Se NPs applied (mg L^−1^) plus 50 mM of NaCl. FW: Fresh weight. Different lowercase letters per bar indicate significant differences according to Fisher’s least significant difference test (*p* < 0.05). Different capital letters per treatment indicate significant differences according to the Hotelling test (*p* < 0.05).

The results were consistent with respect to chlorophyll content; in all cases, the treatments with an application of Se NPs were better than the control (Figure 2). In addition, treatment with 20 mg L^−1^ of Se NPs + NaCl produced the highest content of chlorophylls a and b, and, in total, were 166%, 224%, and 193% more than that of the control, respectively, while compared to the NaCl-only treatment, they were 71%, 120%, and 97% higher, respectively. The MANOVA confirmed the results; considering the three variables of photosynthetic pigments, the best treatment was 20 mg L^−1^ of Se NPs + NaCl. In addition, all of the treatments with Se NPs were found to be better than the control.

### 2.3. Non-Enzymatic Antioxidant Compounds

**Figure 3:**
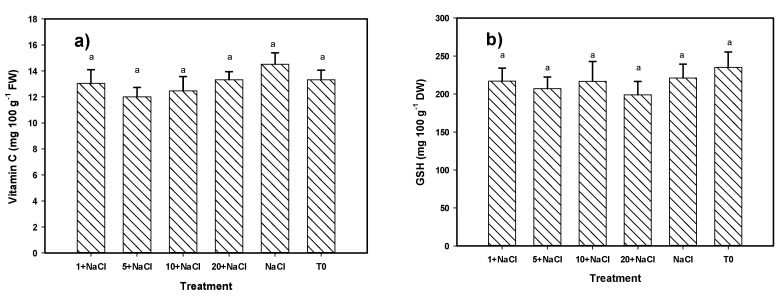

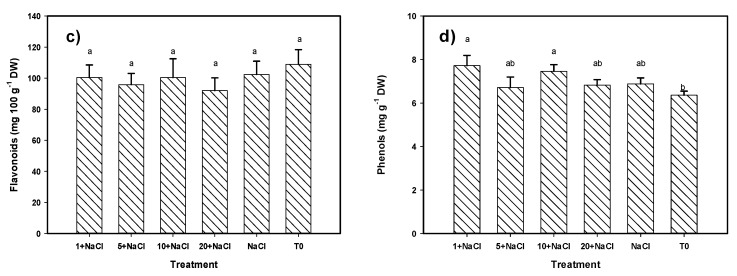
Non-enzymatic antioxidant compounds (**a**) Vitamin C, (**b**) GSH, (**c**) Flavonoids, (**d**) Phenols) in tomato leaves under NaCl stress and an application of Se NPs. T0: Control. NaCl: NaCl-only treatment. 1, 5, 10, and 20 + NaCl: Represent the amount of Se NPs applied (mg L^−1^) plus 50 mM of NaCl. FW: Fresh weight. DW: Dry weight. Different letters per bar indicate significant differences according to Fisher’s Least Significant Difference test (*p* < 0.05).

The content of non-enzymatic antioxidant compounds in leaves was only different in phenols, while there were no significant differences in vitamin C, glutathione, and flavonoids (Figure 3). The content of phenols in the leaves was greater with the treatments of 1 and 10 mg L^−1^ of Se NPs + NaCl, being 21% and 17% more than that with the control, respectively. Considering all of the non-enzymatic antioxidant compound variables in leaves, no significant differences were detected among treatments according to the MANOVA (Table 2).

**Figure 4:**
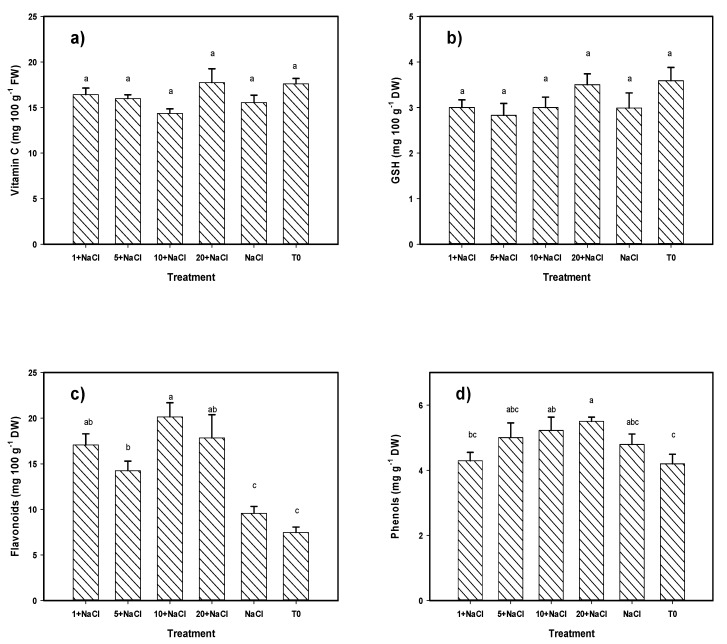

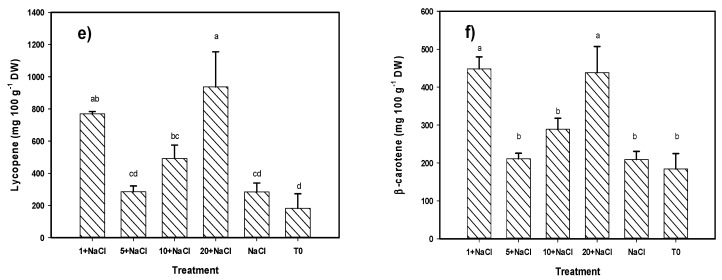
Non-enzymatic antioxidant compounds (**a**) Vitamin C, (**b**) GSH, (**c**) Flavonoids, (**d**) Phenols, (**e**) Lycopene, (**f**) β-carotene) in tomato fruits under NaCl stress and an application of Se NPs. T0: Control. NaCl: NaCl-only treatment. 1, 5, 10, and 20 + NaCl: Represent the amount of Se NPs applied (mg L^−1^) plus 50 mM of NaCl. FW: Fresh weight. DW: Dry weight. Different letters per column indicate significant differences according to Fisher’s least significant difference test (*p* < 0.05).

Regarding the content of non-enzymatic antioxidant compounds in fruit, the results showed significant differences with respect to the control in the content of flavonoids, phenols, lycopene, and β-carotene (Figure 4). Regarding the content of vitamin C and glutathione, no significant differences were observed among treatments. The content of flavonoids increased with the application of Se NPs, since all of the treatments exceeded both the control and the NaCl-only treatment. The best treatment was 10 mg L^−1^ of Se NPs + NaCl, being 169% higher than the control and 110% higher than the NaCl-only treatment. Regarding the content of phenols, only the treatments of 20 and 10 mg L^−1^ of Se NPs + NaCl generated a positive effect; these produced a 30% and 24% higher phenol content than the control, respectively. The lycopene content was increased with the treatment of 20 mg L^−1^ of Se NPs + NaCl by 414% with respect to the control and 229% in comparison to the NaCl-only treatment. The treatment of 1 mg L^−1^ of Se NPs + NaCl was also higher than the control and the NaCl-only treatment by 322% and 172%, respectively. The treatments of 1 and 10 mg L^−1^ of Se NPs + NaCl also increased the content of β-carotene in relation to the control and the NaCl-only treatment by 138–143% and 109–114%, respectively.

Considering the effect of all non-enzymatic antioxidant compounds in fruits that were identified from the MANOVA, it can clearly be observed that the treatment of 20 mg L^−1^ of Se NPs + NaCl was the best, followed by the treatment of 1 mg L^−1^ of Se NPs + NaCl, both being better than the control and the NaCl-only treatment (Table 2).

### 2.4. Enzymatic Antioxidant Compounds

**Figure 5:**
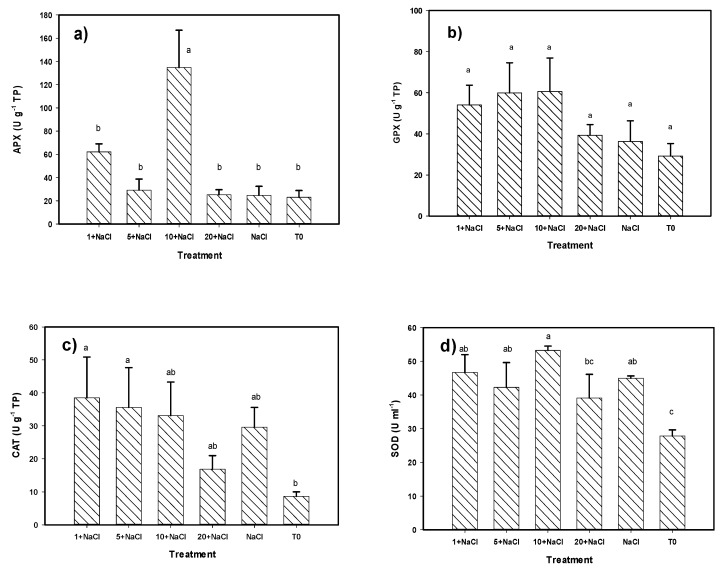
Enzymatic antioxidant compounds (**a**) APX, (**b**) GPX, (**c**) CAT, (**d**) SOD in tomato leaves under NaCl stress and an application of Se NPs. T0: Control. NaCl: NaCl-only treatment. The terms 1, 5, 10, and 20 + NaCl represent the amount of Se NPs applied (mg L^−1^) plus 50 mM of NaCl. APX: Ascorbate peroxidase. GPX: Glutathione peroxidase. CAT: Catalase. SOD: Superoxide dismutase. TP: Total proteins. Different letters per bar indicate significant differences according to Fisher’s least significant difference test (*p* < 0.05).

The results of the enzymatic activity analysis of ascorbate peroxidase (APX), catalase (CAT), and superoxide dismutase (SOD) show significant differences of the treatments with Se NPs with respect to the control in the leaves of the tomato plants (Figure 5). Regarding APX and SOD, the best treatment was 10 mg L^−1^ of Se NPs + NaCl; 486% and 91% higher than the control, respectively. The treatments of 1 and 5 mg L^−1^ of Se NPs + NaCl increased the SOD activity with respect to the control by 68% and 52%, respectively, and the NaCl-only treatment was better than the control by 61%. No significant differences were observed between the Se NPs + NaCl treatments and the NaCl-only treatment. The CAT activity was only increased with the treatments of 1 and 5 mg L^−1^ of Se NPs + NaCl with respect to the control, by 345% and 312%, respectively. Considering all of the enzymatic variables, the best treatment was 10 mg L^−1^ of Se NPs + NaCl, which was better than both the control and the NaCl-only treatment, followed by 1 mg L^−1^ of Se NPs + NaCl, which was only better than the control.

**Figure 6:**
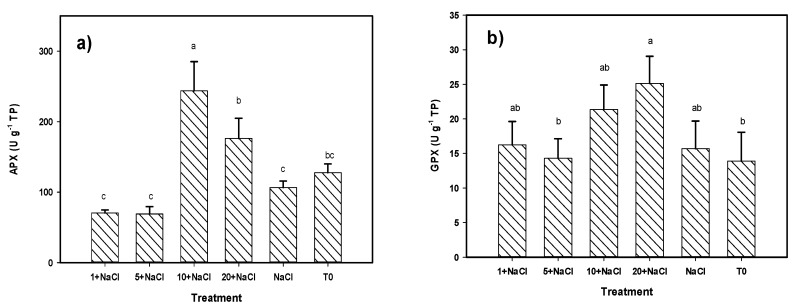

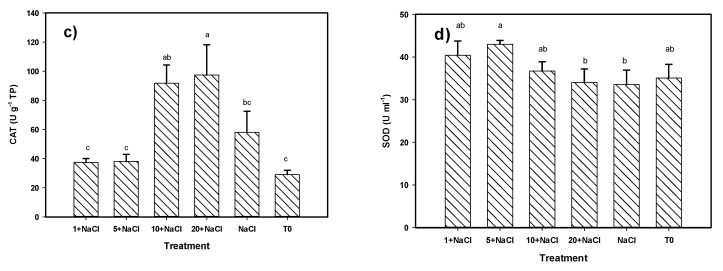
Enzymatic antioxidant compounds (**a**) APX, (**b**) GPX, (**c**) CAT, (**d**) SOD in tomato fruits under NaCl stress and an application of Se NPs. T0: Control. NaCl: NaCl-only treatment. The terms 1, 5, 10, and 20 + NaCl represent the amount of Se NPs applied (mg L^−1^) plus 50 mM of NaCl. APX: Ascorbate peroxidase. GPX: Glutathione peroxidase. CAT: Catalase. SOD: Superoxide dismutase. TP: Total proteins. Different letters per bar indicate significant differences according to Fisher’s least significant difference test (*p* < 0.05).

In the fruit, all enzymes showed differences among treatments (Figure 6). The enzymatic activity of APX was greater with the treatment of 10 mg L^−1^ of Se NPs + NaCl by 90% and 129% in comparison to the control and the NaCl-only treatment, respectively. The glutathione peroxidase (GPX) activity was higher in the treatment of 20 mg L^−1^ of Se NPs + NaCl than in the control by 80%. Regarding the CAT activity, the treatment of 20 mg L^−1^ of Se NPs + NaCl was better than that of the control and the NaCl-only treatment by 235% and 67%, respectively. Regarding the SOD activity, the best treatment was 5 mg L^−1^ of Se NPs + NaCl, although it was only better than the NaCl-only treatment by 28%. The MANOVA results that considered the joint effect of the four enzymes indicate that the best treatment was 10 mg L^−1^ of Se NPs + NaCl followed by 20 mg L^−1^ of Se NPs + NaCl, both being better than the rest of the treatments (Table 2).

### 2.5. Fruit Quality

**Figure 7:**
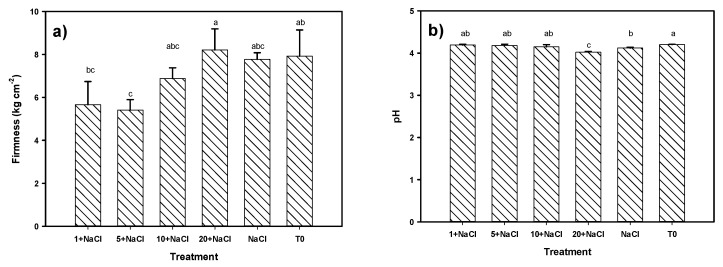

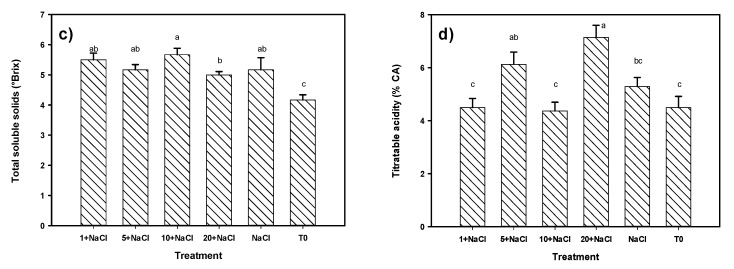
Tomato fruit quality parameters (**a**) Firmness, (**b**) pH, (**c**) Total soluble solids, (**d**) Titratable acidity of plants under NaCl stress and an application of Se NPs. T0: Control. NaCl: NaCl-only treatment. The terms 1, 5, 10, and 20 + NaCl represent the amount of Se NPs applied (mg L^−1^) plus 50 mM of NaCl. Different letters per column indicate significant differences according to Fisher’s least significant difference test (*p* < 0.05).

Regarding fruit quality characteristics, significant differences were observed in the total content of soluble solids, where all of the treatments that included NaCl were better than the control. The treatment of 10 mg L^−1^ of Se NPs + NaCl had the highest concentration (36%) (Figure 7). The rest of the treatments were found to be better than the control in the range of 20–32%. Regarding the titratable acidity of the fruits, the treatment of 20 mg L^−1^ of Se NPs + NaCl was the best, and it was higher than the control by 59% and the NaCl-only treatment by 17%. Regarding the firmness of the fruits, the treatment with 20 mg L^−1^ of Se NPs + NaCl was the best. However, it was not significantly different to the control or the NaCl-only treatment; on the contrary, the treatment with 5 mg L^−1^ of Se NPs + NaCl produced fruits with the least firmness. Regarding the pH of the fruit, the control was the highest, and we observed a tendency for the pH to decrease as the number of NPs increased. Hence, the treatment of 20 mg L^−1^ of Se NPs + NaCl had the lowest pH, being 4.3% lower than the control.

## 3. Discussion

### 3.1. Crop Growth

Tomato plants that are grown in high concentrations of salt show reduced growth, fruit size, and fruit yield. High concentrations of salt also affect the physiology and morphology of tomato plants [18], as observed in the results of the present study. It has also been reported that NPs have a positive influence on various physiological aspects of, as well as produce greater root and stem growth in, horticultural crops [19]. Hernández-Hernández et al. [20] report that Cu NPs had a positive effect on tomato yield under conditions of salt stress. On the other hand, El-Batal et al. [21] mention that the use of Ag NPs and K_2_SO_4_ had a growth promotion effect in potato plants, and that the addition of Se NPs and ascorbic acid produced an additional growth promotion effect. Positive effects can be induced by NPs when applied at low concentrations, and these effects have been shown to promote the growth of potato plants when they are under stress [21]. Likewise, Domokos-Szabolcsy et al. [22] report that the application of Se NPs (265–530 μM) stimulated the organogenesis and the growth of the root system in a tobacco tissue culture (*Nicotiana tabacum*) in vitro. This could explain the increase in the weight of fruits per plant and fresh weight of aerial biomass that we observed in comparison to the control, which was subjected to NaCl, since the results are consistent with those of the abovementioned studies.

### 3.2. Photosynthetic Pigments

Chlorophylls are very important in plants because they control their photosynthetic potential by capturing the light energy of the sun, and they are the most important photosynthetic pigments [23]. Therefore, chlorophyll content is a key indicator of the photosynthetic capacity of plants [24]. As Hernández-Hernández et al. [20] showed, salinity stress increases the amount of chlorophylls; however, the authors did not find an increase in content with an application of Cu NPs. Abbas [25] reports that selenium increased the content of chlorophyll a and b and in total, and proposes that the change in the level of these photosynthetic pigments is related to the effect of selenium ions on the state of reduction of the oxidation of the leaves. This coincides with the results of this research, where an increase in photosynthetic pigments with the application of Se NPs was clearly observed. The observed effect was increased by the particular characteristics of the NPs that were present, which allowed them to move through the plant more easily [8]. Therefore, the application of Se NPs may help to maintain the photosynthetic capacity of tomato plants under conditions of NaCl stress.

### 3.3. Non-Enzymatic Antioxidant Compounds

Lycopene and β-carotene are the largest natural antioxidants in tomato fruits, the most important carotenoids, and act as inhibitors of singlet oxygen [26]. In terms of human health, lycopene protects cells from oxidative stress caused by free radicals [27]. The application of NPs has been shown to increase the content of these compounds in tomato. Raliya et al. [28] mention that the application of ZnO and TiO_2_ NPs to soil increased the lycopene content in tomato. In addition, Hernández-Hernández et al. [20] mention that, under conditions of stress due to the fact of salinity, the application of Cu NPs increased the content of antioxidant compounds, including lycopene, in tomato fruits. Hernández-Fuentes et al. [29] report an increase in the content of antioxidant compounds, including vitamin C, β-carotene, and phenols, in tomato fruits after applying Cu NPs. Therefore, our observed increase in these compounds in tomato fruits can be attributed to the use of Se NPs, which is relevant since it can increase the capacity to tolerate oxidative stress and thereby benefit human health.

Phenols are antioxidants that give rise to a series of secondary metabolites that are synthesized through the shikimic acid or malonic acid pathways and can exert cellular signaling functions under conditions of abiotic stress [30,31]. Flavonoids can also act as antioxidants that protect plants from oxidative stress by eliminating H_2_O_2_ and singlet oxygen generated under conditions of biotic or abiotic stress [32]. Therefore, an increase in these types of compounds can help to reduce oxidative stress in tomato plants. As has been reported in several studies, the use of NPs can contribute to the production of antioxidant compounds in plants [33], which is in agreement with the results that were obtained in this work. In addition to this, selenium by itself has the ability to increase the production of antioxidant compounds in plants [13], since it is a cofactor in antioxidant enzymes such as glutathione peroxidases [34]. This capacity can be enhanced when applied in the form of NPs due to the physicochemical characteristics that they possess [9] and with the advantage that they have low toxicity [15]. Application of Cu NPs has been shown to increase the amount of antioxidant compounds, such as phenols and flavonoids, in tomato fruits, in addition to vitamin C and lycopene [35]. However, the authors mention that high doses (500 mg L^−1^) of these NPs can cause adverse effects, so it is important to consider the doses to be used. The abovementioned results are consistent with those observed in the present study, which indicates that the use of Se NPs can help to increase the production of antioxidant compounds in tomato plants and, therefore, the benefits that tomato fruits provide to the human diet.

### 3.4. Enzymatic Antioxidant Compounds

The harmful effects of reactive oxygen species (ROS) on plant cells are suppressed by the action of antioxidant enzymes, such as APX, CAT, GPX, and SOD, that scavenge ROS [36]. It is well-documented that the use of NPs can modify the activity of antioxidant enzymes in plants, generally positively, due to the ability to activate the antioxidant defense system [33]. Barrios et al. [37] mention that, after applying CeO_2_ NPs coated with citric acid, the activity of the CAT enzyme in tomato leaves increased. Salinity stress causes an overproduction of ROS, which can cause damage due to the oxidative stress in the cells [5]. However, it has been reported that the application of Cu NPs can help tomato plants tolerate salinity stress due to the regulation of oxidative and ionic stress, possibly due to the expression of the *SOD* and *JA* (jasmonic acid) genes [38]. In addition, it has been demonstrated that the application of Cu NPs increased the activity of the enzymes APX, GPX, CAT, and SOD in tomato plants [38]. Djanaguiraman et al. [39] suggest that the acquisition of tolerance to abiotic stress is closely related to the elimination of ROS, so our observed increase in the activity of antioxidant enzymes may be an indicator of a decrease in salinity stress in the leaves and fruits of tomato plants.

### 3.5. Fruit Quality

The total content of soluble solids in fruits is important due to the potential changes in their organoleptic properties. In addition to this, the quality of the fruit is highly influenced by the pH, since less acidic fruits have a better flavor and are, therefore, more appreciated by consumers [40]. In a natural way, the ripening of the fruit generates a greater accumulation of sugars, such as fructose and glucose, which contribute to the total content of soluble solids in the fruits [41]. According to our results, salinity stress may have induced an advanced maturation of the fruits that resulted in an increase in the total content of soluble solids.

The observed decrease in the pH of the tomato fruits may be due to the accumulation of organic acids in the vacuoles [42]. This is in agreement with Pinedo-Guerrero et al. [43], who reported that an application of Cu NPs + chitosan in a jalapeño pepper crop diminished the pH of the fruits. However, the application of Cu NPs in a tomato culture was shown to induce an increase in the pH in the fruits. This indicates that the effect of NPs can vary depending on their type and the plant species in which they are being evaluated. In addition to this, salinity stress may have direct effects on the quality of fruits as it can reduce the concentration of minerals, such as Ca and K [4], in conjunction with the effect of reactive oxygen species [5].

Juárez-Maldonado et al. [11] report an increase in the titratable acidity of tomato fruits after an application of Cu NPs + chitosan. In addition, Pinedo-Guerrero et al. [43] also observed an increase in the titratable acidity of jalapeño pepper fruits after Cu NPs + chitosan-PVA (Polyvinyl alcohol) were applied. These results are consistent with those of the present study, where an increase in the titratable acidity of tomato fruits was observed after the application of Se NPs. Therefore, application of Se NPs could increase the amount of organic acids in tomato fruits.

## 4. Materials and Methods

### 4.1. Crop Development

The experiment consisted in developing a tomato crop (of the indeterminate growth tomato type saladette “El Cid F1”) under salinity conditions by NaCl and with an application of selenium nanoparticles. The crop was established under greenhouse conditions in a multi-tunnel greenhouse with a polyethylene cover, an average photosynthetically active radiation of 1000 μmol m^−2^ s^−1^, a temperature of 32 °C, and a relative humidity of 60%. A soil-less cultivation system with a mixture of peat moss and perlite as a substrate in a 1:1 ratio (*v*/*v*) was used. The plants were placed in 10 L polyethylene bags and irrigated with Steiner’s nutritive solution [44] using a directed irrigation system. The crop was developed for 102 days and was managed according to conventional agricultural practices.

**Figure 8:**
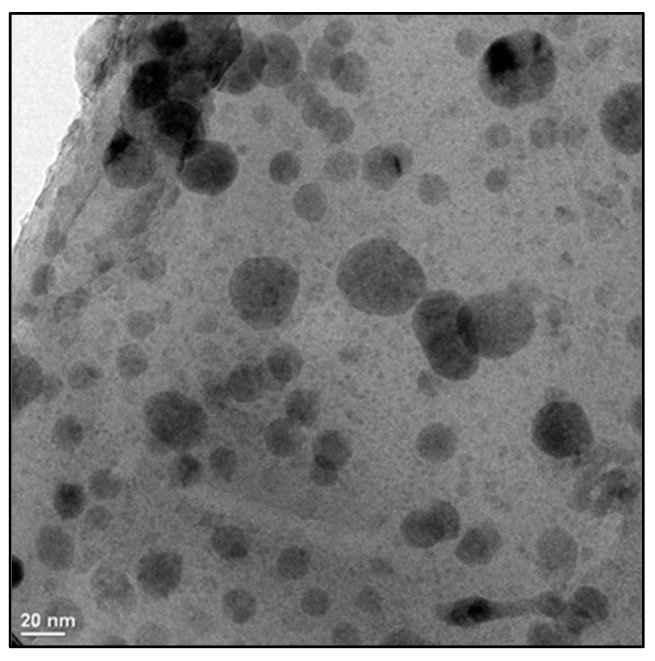
A TEM image of the selenium nanoparticles.

The treatments consisted of an application of selenium nanoparticles to the crop, being the following: a control without stress or Se NPs (T0); an NaCl-only treatment; and four concentrations of Se NPs based on Safari et al. [45] (1, 5, 10, and 20 mg L^−1^) plus stress by salinity. Sodium chloride (NaCl) was applied directly to the nutrient solution at a concentration of 50 mM throughout the development of the crop. This concentration of NaCl was selected based on previous works [20,46]. In the treatments with Se NPs, five applications (2.5, 5.5, 13, 17.1, and 17.1 mL per application, respectively, adding a total of 55.2 mL per plant) of solution were performed with the abovementioned concentrations of Se NPs directly to the substrate every two weeks, starting at 11 days after the transplant. The selenium nanoparticles were synthesized in the Research Center of Applied Chemistry (Saltillo, Mexico) by the procedure of Kong et al. [47]; however, instead of using Arabic gum as a stabilizer, we used chitosan (CS)-PVA as described in Quiterio-Gutiérrez et al. [48]. The NPs were of a spherical shape and had a size of 2–20 nm, as can be seen in Figure 8. The Se NPs had a zeta potential of −29.4 mV. This value suggests that the Se NPs were well-dispersed in the CS-PVA solution.

### 4.2. Sample Processing

Samples of leaf tissue were collected, random plants were selected, and four fully expanded young leaves were taken for a biochemical analysis. Also, uniformly sized fruits were collected at Stage 6 (light red) of maturity. Some samples were used to perform a determination in fresh tissue immediately, and the other samples were stored at −20 °C and then lyophilized as described in López-Vargas et al. [35].

### 4.3. Photosynthetic Pigments

The content of chlorophyll (mg 100 g^−1^ fresh weight (FW)) was determined using the method of Nagata and Yamashita [49]. For this, the absorbance at 645 and 663 nm was determined and used in Equations (1) and (2). Total chlorophyll (Chl) was considered to be the sum of Chl a and Chl b.

(1) $$\mathrm{Chl}\;a=0.999\times {\mathrm{Abs}}_{663}-0.0989\times {\mathrm{Abs}}_{645}$$

(2) $$\mathrm{Chl}\;b=-0.328\times {\mathrm{Abs}}_{663}+1.77\times {\mathrm{Abs}}_{645}$$

### 4.4. Enzymatic and Non-Enzymatic Antioxidants

The quantification of total proteins (mg g^−1^ of dry weight (DW)) was performed using Bradford’s colorimetric technique [50], in a microplate, 5 μL of the extract and 250 μL of Bradford reagent were placed in each well. They were incubated for 10 min at room temperature (26 °C), and then read at a wavelength of 630 nm on a microplate reader (BioTek, ELx808 model, Winooski, VT, USA).

Catalase (CAT) (QE 1.11.1.6) (U TP^−1^, where U is equal to the mM equivalent of H_2_O_2_ consumed per milliliter per minute) was quantified by the method of Dhindsa et al. [51]. The measurement was carried out in two steps (at time 0 (T0) and at time 1 (T1)). At T0, 100 μL of extract, 400 μL of H_2_SO_4_ (5%), and 1 mL of H_2_O_2_ (100 mM) were added to an Eppendorf tube and vortexed for 30 s. The absorbance was then measured on a UV-Vis spectrophotometer (Thermo Fisher Scientific, G10S model, Waltham, MA, USA) with a quartz cell at 270 nm. At T1, 100 μL of extract and 1 mL of H_2_O_2_ (100 μL) were added and stirred for 1 min in a vortex at 26 °C. Then, 400 μL of H_2_SO_4_ (5%) was added to stop the reaction and the absorbance was measured by a UV-Vis spectrophotometer (Thermo Fisher Scientific, G10S model, Waltham, MA, USA) with a quartz cell at 270 nm. The determination of catalase was based on the quantification of the oxidation rate of H_2_O_2_ by absorbance difference (T0–T1).

Superoxide dismutase (SOD) (QE 1.15.1.1) (U mL^−1^, where U is defined as the amount of enzyme needed to exhibit 50% dismutation of the superoxide radical) was carried out using the SOD Cayman 706002^®^ kit. A mix of 20 μL of extract, 200 μL of the radical detector (tetrazolium salt), and 20 μL of xanthine oxidase solution was placed in a microplate. The microplate was covered with a transparent cover (kit), stirred for 10 s, and then incubated at 26 °C for 30 min. The absorbance was then measured at a length of 450 nm using a plate reader (BioTek, ELx808 model, Winooski, VT, USA). The principle of the test was based on the use of a tetrazolium salt for the detection of superoxide radicals generated by xanthine oxidase and hypoxanthine.

Glutathione peroxidase (GPX) (QE 1.11.1.9) (U TP^−1^, where U is equal to the mM equivalent of reduced glutathione (GSH) per milliliter per minute) was determined by the method of Flohé and Günzler [52]. A mix of 200 μL of extract, 400 μL of GSH (0.1 mM), and 200 μL of Na_2_HPO_4_ (0.067 M) was placed in a test tube. The mixture was preheated in a water bath at 25 °C for 5 min, then 200 μL of H_2_O_2_ (1.3 mM) was added to start the catalytic reaction for 10 min at a temperature of 26 °C. The reaction was stopped by the addition of 1 mL of 1% trichloroacetic acid. The mixture was placed in an ice bath for 30 min, and then centrifuged at 1008× *g* for 10 min at 4 °C. To assess the glutathione peroxidase, 480 μL of the supernatant, 2.2 mL of Na_2_HPO_4_ (0.32 M), and 320 μL of 5,5-dithio-bis-2-nitrobenzoic acid dye (DTNB) of 1 mM were placed in a test tube. The absorbance was measured by a UV-Vis spectrophotometer (Thermo Fisher Scientific, G10S model, Waltham, MA, USA) at 412 nm with a quartz cell.

Ascorbate peroxidase (APX) (QE 1.11.1.1) was determined by the method of Nakano and Asada [53] and is expressed as U per gram of total proteins (U g^−1^ TP), where U is equal to the μmol of oxidized ascorbate per milliliter per minute. The measurement was undertaken at two moments (at time 0 (T0) and at time 1 (T1)). At T0, a mix of 100 μL of extract, 500 μL of ascorbate (10 mg L^−1^), 400 μL of H_2_SO_4_ (5%), and 1 mL of H_2_O_2_ (100 mM) were placed in a test tube, and then vortexed for 30 s. The absorbance was measured in a UV-Vis spectrophotometer (Thermo Fisher Scientific, G10S model, Waltham, MA, USA) at 266 nm with a quartz cell. At T1, 100 μL of extract, 500 μL of ascorbate (10 mg L^−1^), and 1 mL of H_2_O_2_ (100 mM) were added to the previous mixture and vortexed for 1 min at a temperature of 26 °C. To stop the reaction, 400 μL of H_2_SO_4_ (5%) was added, and the absorbance was measured. Ascorbate peroxidase determination was based on the quantification of the ascorbate oxidation rate by means of the absorbance difference (T0–T1).

Lycopene and β-carotene (mg 100 g^−1^ DW) were determined according to Nagata and Yamashita [49], using the absorbance values of 453, 505, 645, and 663 nm in Equations (3) and (4).

(3) $$\mathrm{Lycopene}=-0.0458\times {\mathrm{Abs}}_{663}+0.204\times {\mathrm{Abs}}_{645}+0.372\times {\mathrm{Abs}}_{505}-0.0806\times {\mathrm{Abs}}_{453}$$

(4) $$\beta -\mathrm{carotene}=0.216\times {\mathrm{Abs}}_{663}-1.22\times {\mathrm{Abs}}_{645}-0.304\times {\mathrm{Abs}}_{505}+0.452\times {\mathrm{Abs}}_{453}$$

Vitamin C (mg 100 g^−1^ FW) was determined by the colorimetric method using 2,6 dichlorophenol, 1 g of fresh tissue and HCl (2%), as described in Padayatt et al. [54].

Glutathione (mg 100 g^−1^ DW) was determined using the method of Xue et al. [55] by means of a 5,5-dithio-bis-2 nitrobenzoic acid (DTNB) reaction. A mix of 0.480 mL of the extract, 2.2 mL of sodium dibasic phosphate (Na_2_HPO_4_ at 0.32 M), and 0.32 mL of the DTNB dye (1 mM) was placed in a test tube. Then, the mix was vortexed and read on a UV-Vis spectrophotometer (Thermo Fisher Scientific, G10S model, Waltham, MA, USA) at 412 nm using a quartz cell.

Flavonoids (mg 100 g^−1^ DW) were determined by the method of Arvouet-Grand et al. [56]. For the extraction, 100 mg of lyophilized tissue was placed in a test tube, where 10 mL of reagent grade methanol was added and shaken for 30 s until the mixture was homogenized. The mixture was filtered using No. 1 Whatman paper. For the quantification, 2 mL of the extract and 2 mL of methanolic solution of aluminum trichloride (AlCl_3_) 2% were added to a test tube and left to rest for 20 min in the dark. The reading was then taken in a UV-Vis spectrophotometer (Thermo Fisher Scientific, G10S model, Waltham, MA, USA) at a wavelength of 415 nm using a quartz cell.

Phenols (mg 100 g^−1^ DW) were determined with Folin–Ciocalteu reagent, as described in Cumplido-Nájera et al. [57]. The sample (0.2 g) was extracted with 1 mL of a water:acetone solution (1:1). The mixture was vortexed for 30 s. The tubes were centrifuged (Thermo Scientific Mod. ST 16R centrifuge, Langenselbold, Germany) at 17,500× *g* for 10 min at 4 °C. In a test tube, 50 μL of the supernatant, 200 μL of the Folin–Ciocalteu reagent, 500 μL of 20% sodium carbonate (Na_2_CO_3_), and 5 mL of distilled water were added and then vortexed for 30 s. The samples were placed in a water bath at 45 °C for 30 min. Finally, the reading was taken at an absorbance of 750 nm using a plastic cell in a UV-Vis spectrophotometer (Thermo Fisher Scientific, G10S model, Waltham, MA, USA).

### 4.5. Fruit Quality

The parameters that describe a fruit’s quality (hydrogen potential (pH), total soluble solids (TSS), fruit firmness, and titratable acidity (TA)) were determined as described in López-Vargas et al. [35]. For this, six fruits (one per plant) of uniform size and in a light red state of maturity were collected from the third cluster.

### 4.6. Statistical Analysis

Six replicates per treatment were considered for each of the evaluated variables in a completely random design. Each replicate was obtained from a different plant. An analysis of variance and Fisher’s least significant difference mean test (*p* < 0.05) were applied to each variable. Moreover, crop growth, photosynthetic pigment, and non-enzymatic and enzymatic antioxidant data were analyzed by a multivariate analysis of variance (MANOVA). Post-hoc multiple comparisons among multivariate means of treatments were performed by the Hotelling test (*p* < 0.05). All statistical processes were performed using the software Infostat 2018 [58].

## 5. Conclusions

The results of the present study show that the application of selenium nanoparticles improved the yield and aerial biomass of tomato plants that were developed under saline stress conditions, partly due to the presence of an increase in the amount of photosynthetic pigments in the leaves, which can improve the photosynthetic capacity of the plants.

The selenium nanoparticles were found to increase the concentration of phenols in the leaves. However, they also increased the majority of the antioxidant compounds that were present in the tomato fruits (lycopene, β-carotene, flavonoids, and phenols). This result represents an additional advantage for the production of tomato under salt stress, since the plants will produce fruits of better quality that may benefit the human diet.

The application of selenium nanoparticles was found to increase the enzymatic activity in leaves and fruits of the tomato plants; therefore, Se NPs can increase the capacity of tomato plants to tolerate NaCl stress.

The application of selenium nanoparticles diminished the effect of salinity on tomato plants due the fact of its stimulatory effect on growth and the antioxidant defense system; however, it is necessary to carry out more studies in order to identify the salinity levels in which a better effect can be observed. In addition, we recommend that further studies be carried out on these nanoparticles with the aim of increasing the nutraceutical characteristics and bio-fortification of fruits, since better results might be obtained with objectives other than inducing tolerance to salinity stress.

## Figures and Tables

**Table 1:** Growth variables of tomato plants under NaCl stress and an application of Se nanoparticles (NPs).

Treatment	PH (cm)	SD (mm)	NL	NC	NF	AFW (g)	FW (kg)	FAB (kg)	DAB (g)	MANOVA
T0	2.34 ^a^	11.96 ^a^	28.38 ^b^	7.24 ^a^	51.43 ^a^	102.34 ^a^	5.24 ^a^	1.91 ^a^	136.7 ^a^	7018 ^† ‡ A^
NaCl	2.22 ^b^	11.44 ^ab^	29.75 ^a^	7.40 ^a^	43.55 ^c^	76.26 ^b^	3.35 ^c^	1.38 ^c^	94.6 ^b^	4996 ^BC^
1 + NaCl	2.22 ^b^	10.98 ^b^	28.86 ^ab^	7.29 ^a^	45.29 ^bc^	78.55 ^b^	3.55 ^bc^	1.32 ^c^	90.2 ^c^	5136 ^BC^
5 + NaCl	2.19 ^b^	11.43 ^ab^	28.71 ^ab^	7.14 ^a^	42.62 ^c^	76.49 ^b^	3.27 ^c^	1.30 ^c^	88.7 ^c^	4831 ^C^
10 + NaCl	2.15 ^b^	11.43 ^ab^	28.57 ^b^	7.33 ^a^	48.38 ^ab^	78.17 ^b^	3.78 ^b^	1.55 ^b^	108.4 ^b^	5615 ^B^
20 + NaCl	2.16 ^b^	11.46 ^ab^	29.00 ^ab^	7.48 ^a^	46.43 ^abc^	79.09 ^b^	3.66 ^bc^	1.34 ^c^	91.8 ^c^	5266 ^BC^
CV (%)	7.1	9.9	6.1	8.4	17.9	11.5	20.7	19.2	21.9	*p* < 0.0001

T0: Control. NaCl: NaCl-only treatment. 1, 5, 10, and 20 + NaCl: Represent the amount of Se NPs applied (mg L^−1^) plus 50 mM of NaCl. CV: Coefficient of variation. PH: Plant height. SD: Stem diameter. NL: Number of leaves. NC: Number of clusters. NF: Number of fruits. AFW: Average of fruit weight. FW: Fruit weight per plant. FAB: Fresh aerial biomass. DAB: Dry aerial biomass. Different letters per column indicate significant differences according to Fisher’s least significant difference test (*p* < 0.05). ^†^ Data obtained from a matrix of linear correlations of nine response variables from the MANOVA. ^‡^ Different letters indicate significant differences according to the Hotelling test (*p* < 0.05).

**Table 2:** MANOVAs of different variables of leaves and fruits of tomato plants under NaCl stress and an application of Se NPs.

	Non-Enzymatic Antioxidants		Enzymatic Antioxidants	
Treatment	Leaves	Fruits	Leaves	Fruits
T0	363.7 ^† ‡ A^	224.3 ^† ‡ D^	88.6 ^† ‡ C^	205.8 ^† ‡ C^
NaCl	344.8 ^A^	327.8 ^CD^	135.6 ^BC^	213.8 ^C^
1 + NaCl	338.3 ^A^	833.2 ^AB^	201.4 ^AB^	164.6 ^C^
5 + NaCl	321.6 ^A^	334.0 ^CD^	167.0 ^BC^	164.4 ^C^
10 + NaCl	337.2 ^A^	548.9 ^BC^	281.8 ^A^	393.8 ^A^
20 + NaCl	311.1 ^A^	1003.6 ^A^	120.5 ^BC^	332.9 ^B^
	*p* = 0.8382	*p* < 0.0001	*p* = 0.0016	*p* < 0.0001

T0: Control. NaCl: NaCl-only treatment. The terms denoted as 1, 5, 10, and 20 + NaCl represent the amount of Se NPs applied (mg L^−1^) plus 50 mM of NaCl. ^†^ Data obtained from a matrix of linear correlations of the response variables from the MANOVA. ^‡^ Different letters indicate significant differences according to a Hotelling test (*p* < 0.05).
